# Unveiling Primary Bone Tumors of the Spine: A Review of Essential Imaging Clues

**DOI:** 10.3390/diagnostics15232970

**Published:** 2025-11-23

**Authors:** Noah Tregobov, Michal Krolikowski, Ryan Dragoman, Benjamin Brakel, Peter L. Munk, Manraj K. S. Heran

**Affiliations:** 1 Department of Radiology, Faculty of Medicine, University of British Columbia, Vancouver, BC V5Z 1M9, Canada; 2 Vancouver-Fraser Medical Program, Faculty of Medicine, University of British Columbia, Vancouver, BC V5Z 1M9, Canada; 3 Division of Neuroradiology, Department of Radiology, Vancouver General Hospital, University of British Columbia, Vancouver, BC V5Z 1M9, Canada

**Keywords:** primary spinal bone tumors, osseous, imaging, diagnostics, benign, malignant, osteoid, musculoskeletal tumors, computed tomography, magnetic resonance imaging

## Abstract

Primary spinal osseous tumors are relatively rare, comprising ~5–10% of spinal bone neoplasms, whereas metastases account for the vast majority of spinal lesions. Patients commonly present with insidious back pain, sometimes with a focal mass, and constitutional symptoms are uncommon early in the disease course. As clinical features are often nonspecific and may overlap with degenerative, infectious, and metastatic disease, imaging plays an important role in lesion identification, characterization, and treatment planning. Computed tomography helps to define osseous architecture and matrix characteristics. Magnetic resonance imaging can assess marrow involvement, soft tissue extension, neural compression and intra-canal disease, and tumor vascularity. Together, advanced imaging modalities guide further workup, optimize biopsy planning, inform prognostic assessment and therapeutic decision-making, and anticipate mechanical instability or neural compromise. This narrative pictorial review synthesizes radiographic, CT, and MRI appearances of primary spinal tumors across major histologic lineages (e.g., osteogenic, chondrogenic, notochordal, vascular), illustrated with representative cases. We correlate imaging with clinical presentation to distinguish typical from atypical variants and highlight mimics and pitfalls with implications for diagnostic interpretation and management.

## 1. Introduction

The vertebral column is the most frequent site of osseous metastasis and a common location for metastatic disease overall [1,2,3]. However, primary tumors are uncommon, accounting for fewer than 10% of spinal bone tumors overall [4,5,6]. Primary osseous tumor behavior ranges from benign, to intermediate (including locally aggressive), to malignant, and they can be classified by histologic lineage (e.g., osteogenic, chondrogenic) [5]. Localized back pain is a common presenting symptom and may be accompanied by focal tenderness, swelling, or a palpable mass [6]. Neurologic deficits may arise from epidural extension, foraminal involvement, or pathologic fracture with instability. Systemic or constitutional symptoms are uncommon at initial presentation and are typically associated with advanced or metastatic disease.

As clinical findings can be subtle or nonspecific, especially early on in the disease course, imaging plays a central role in lesion identification and characterization, guiding safe biopsy planning (including to minimize tract seeding), informing treatment selection, and anticipating mechanical instability or neural compression that may threaten neurologic function. Radiography can suggest osseous abnormalities and provide clues to matrix composition. Computed tomography (CT) enables detailed evaluation of cortical and trabecular architecture as well as mineralization patterns; advanced imaging such as positron emission tomography (PET)/CT can demonstrate metabolic activity and identify additional sites of involvement when present. Magnetic resonance imaging (MRI) is sensitive for detecting marrow involvement, soft tissue and epidural/neural foraminal extension, and potential neural compression.

This review summarizes the radiographic, CT, and MRI features of primary spinal osseous tumors, illustrated with representative cases and integrated clinical correlates.

## 2. Imaging Findings

### 2.1. Osteogenic

#### 2.1.1. Axial Enostosis (Bone Island)

Enostoses are benign foci of compact (cortical) bone within cancellous bone. They are almost always asymptomatic and detected incidentally on imaging [7]. They occur across all ages and in both sexes, with no clear environmental associations [7,8].

**Figure 1:**
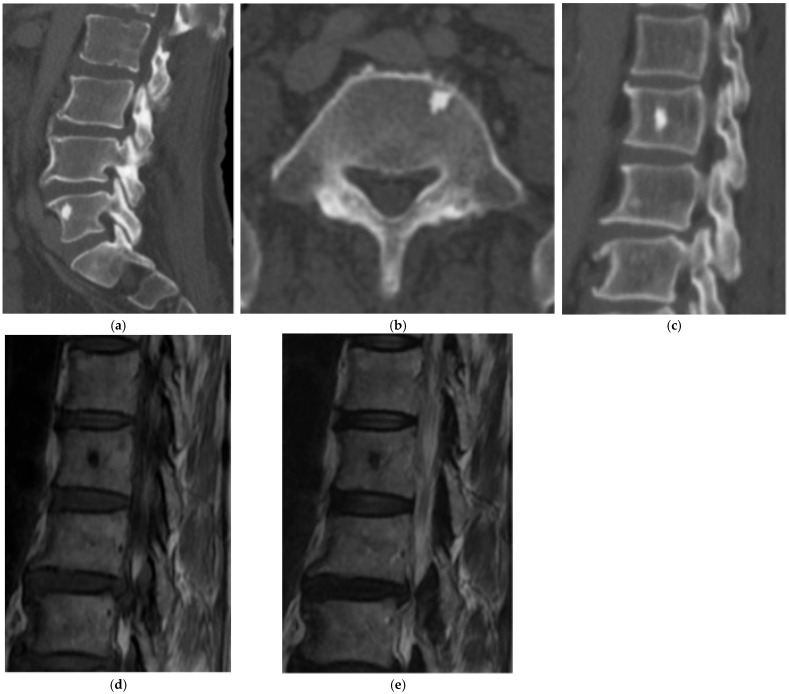
Enostoses/Bone Island. (**a**) Sagittal and (**b**) axial CT bone window demonstrate an enostosis (bone island) in the right anterolateral aspect of the L5 vertebral body. Radiating bony streaks are visualized along the periphery. (**c**) Sagittal CT bone window shows another enostosis in the lumbar spine centered within a vertebral body. (**d**) Sagittal MR T1 and (**e**) T2-weighted MR images show a focus of low signal intensity corresponding to the enostosis.

On radiographs, enostoses appear as round or ovoid, homogeneously dense intraosseous lesions. Clear radiating bony streaks blend with the normal adjacent trabeculae although this is better visualized on CT (Figure 1a–c). These lesions are nonaggressive and there should not be any cortical destruction, periosteal reaction, or soft tissue component. On MRI they are uniformly low signal on T1- and T2-weighted images and typically lack enhancement (Figure 1d,e). Bone scintigraphy is usually non-avid or minimally avid, although rare biopsy-proven cases have shown mild uptake [9]. Compared with osteoblastic metastases, enostoses generally demonstrate higher CT attenuation (often ≳1000 HU) and remain stable on serial imaging [7]. When the imaging appearance is classic on radiographs, CT, and MR, bone scintigraphy should be reserved for atypical cases or when clinical concern for a more aggressive lesion persists. A recent study found spectral CT more accurately differentiates bone enostoses from osteoblastic metastases than plain CT [10].

#### 2.1.2. Osteoid Osteoma

Osteoid osteoma is a benign osteoblastic tumor characterized by a small, well-demarcated nidus (usually <1.5 cm). It occurs most often in adolescents and young adults, with a male predominance [11]. Patients classically report nocturnal pain that improves with NSAIDs or salicylates [12]. Histologically, the nidus comprises disorganized trabecular bone and osteoblasts within a vascular fibrous stroma [11].

**Figure 2:**
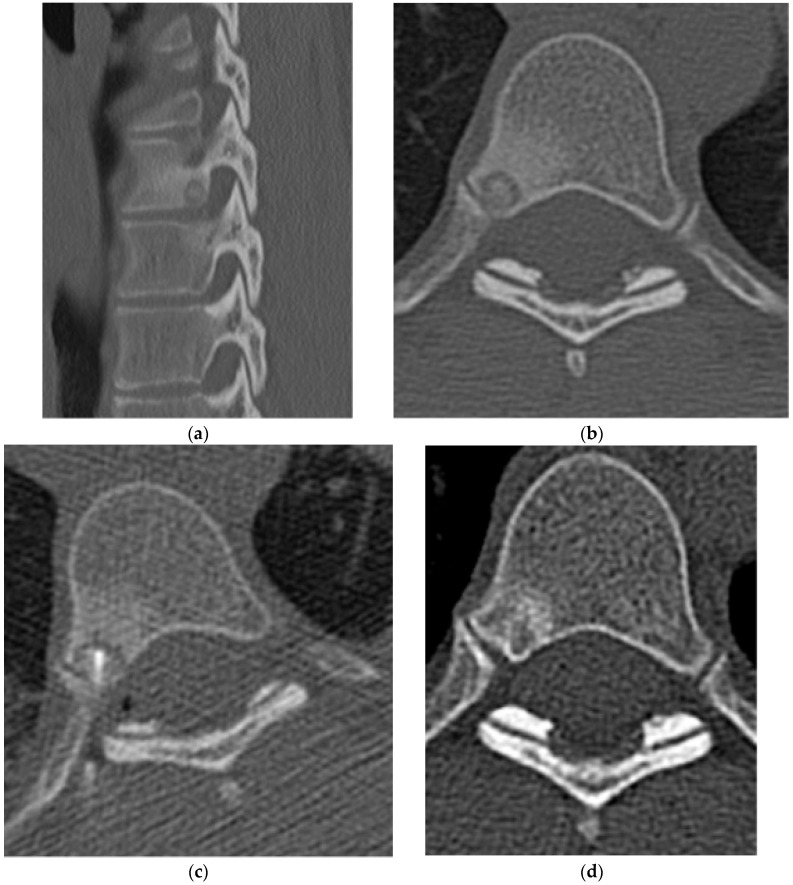
Osteoid osteoma. (**a**) Axial and (**b**) sagittal CT bone window demonstrate an osteoid osteoma in the right posterior aspect of a thoracic vertebral body with a central sclerotic nidus, surrounding lucency, and prominent surrounding edema. (**c**) Axial CT bone window image demonstrates a radiofrequency ablation probe in the nidus. (**d**) Follow-up images show resolution of nidus after radiofrequency ablation treatment.

Imaging mirrors the pathology, with a central nidus and surrounding reactive sclerosis. On CT, the nidus appears as a well-demarcated low attenuation focus that may contain central mineralization, with surrounding reactive sclerosis, which should not be mistaken for a neoplastic process itself (Figure 2a,b). A nidus size <1.5 cm helps distinguish osteoid osteoma from the larger, otherwise similar osteoblastoma. On radiographs, findings parallel CT, but the nidus may be occult with only reactive changes visible. MRI is best used as a problem-solving adjunct because it is sensitive but nonspecific [11]. The reactive marrow edema and hyperemia associated with the lesion also limits MRI evaluation. When visible, the nidus typically shows low-to-intermediate T1 signal and variable T2 signal depending on mineralization [13]. Perilesional edema combined with associated variable enhancement may mimic infection or inflammatory spondylitis, particularly near the disc, with relative disc sparing favoring osteoid osteoma. Although used less commonly, bone scintigraphy can aid pre-procedural localization or evaluation for multiple nidi. The classic imaging appearance is a “double density” sign which represents the central nidus and surrounding reactive sclerosis [11,13]. Although this scintigraphic finding is specific, it is less common in the spine than in the appendicular skeleton [11]. The treatment of choice is thermal ablation with the goal being to destroy the nidus (Figure 2c,d).

#### 2.1.3. Osteoblastoma

Osteoblastoma is a rare, benign osteoblastic neoplasm comprising interconnecting trabeculae lined by osteoblasts within a vascular, loose connective-tissue stroma [14,15]. It accounts for approximately 1% of primary bone tumors. Patients typically present with dull, aching pain that, compared to osteoid osteoma, is less often nocturnal and less reliably relieved by NSAIDS. These tumors can exhibit locally aggressive behavior with bone destruction and soft tissue extension, particularly in the spine [16]. Peak incidence occurs in the second to third decades of life, with a male predominance of roughly 2:1 [14,15].

**Figure 3:**
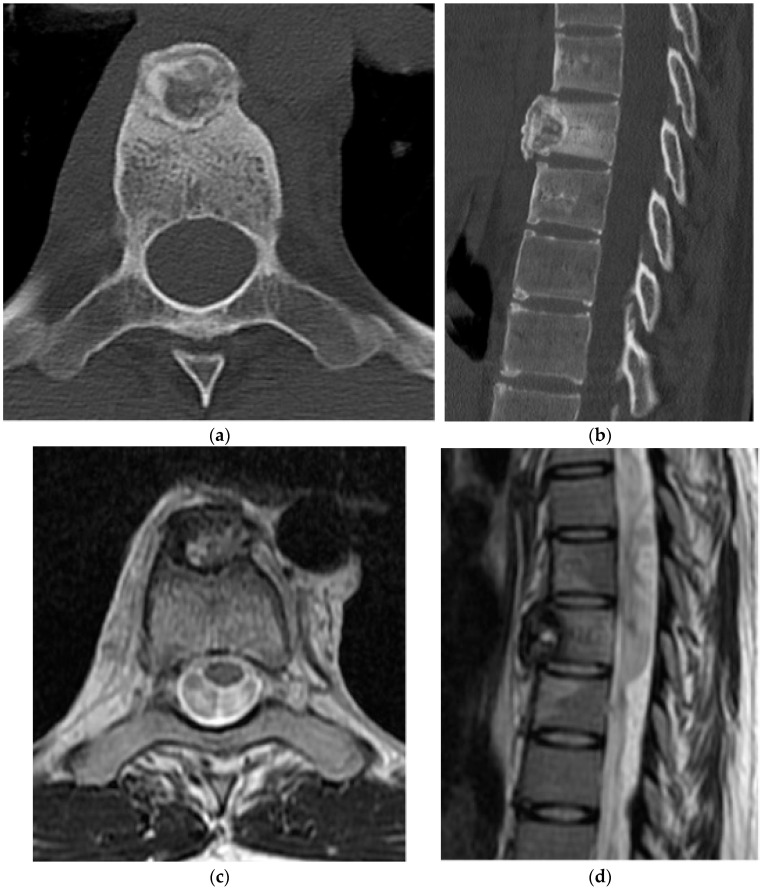
Osteoblastoma. (**a**) Axial and (**b**) sagittal CT bone window, expansile lesion at the anterior margin of T10 with a narrow zone of transition and a central nidus. The lesion measures greater than 1.5 cm, distinguishing it from an osteoid osteoma. Increased sclerosis in the involved vertebral body is due to chronic inflammation. (**c**) Axial and (**d**) sagittal T2 sequences, predominantly T2 hypointense lesion with cortical thickening. There is prominent perilesional edema extending into the adjacent vertebral bodies and the paravertebral soft tissue. This is an atypical location for an osteoblastoma as most are found in the posterior elements (Figure 4).

**Figure 4:**
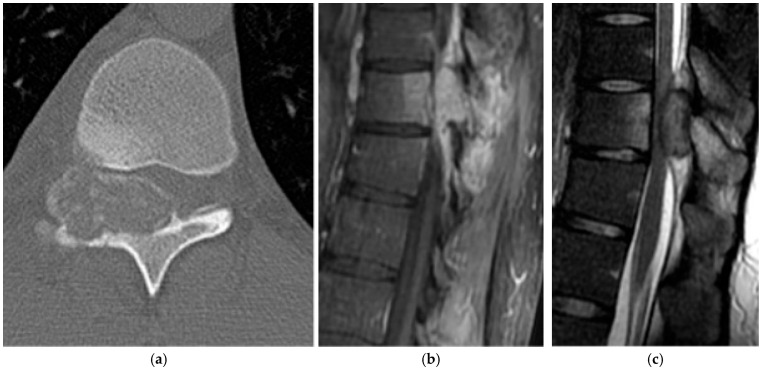
Osteoblastoma. (**a**) Axial CT bone window, expansile lesion extending from the posterior elements of the T11 vertebral body. The cortex is thin but intact. (**b**) Sagittal T1 fat saturation post-contrast MR, avid enhancement in the lesion and surrounding marrow and soft tissue representing perilesional inflammation with associated edema. (**c**) Sagittal T2 MR: the lesion is epidural; the dura is displaced ventrally; perilesional edema is again noted.

On radiographs, osteoblastomas are predominantly lytic, usually >1.5 cm, and may be expansile with variable surrounding sclerosis. A central lucency (sometimes termed a nidus) may be visible on radiograph, but CT is the modality of choice for defining lesion extent and matrix, including central calcification reminiscent of osteoid osteoma (Figure 3a,b and Figure 4a). CT may also demonstrate cortical expansion and remodeling, which, if extensive, can narrow the spinal canal or neural foramina; occasionally, cortical destruction and an associated soft tissue component are present [17].

As with osteoid osteoma, MRI may overestimate lesion size due to reactive marrow edema and enhancement extending into adjacent soft tissues (Figure 3c,d and Figure 4b,c). Osteoblastomas are typically isointense to hypointense on T1- and T2-weighted images due to internal mineralization and show contrast enhancement given their vascularity (Figure 4b) [17]. Enhancement within the nidus or lesion itself is often heterogeneous, sometimes with a peripheral non-enhancing rim demarcating the outer border [17].

Bone scintigraphy typically demonstrates nonspecific avid uptake like other bone-forming neoplasms, limiting diagnostic utility. Hybrid imaging with ^18^F-FDG PET has been proposed as an adjunct in selected spinal cases of osteoblastoma [18]; however, CT and MRI remain the mainstay of evaluation based on current guidelines [19].

#### 2.1.4. Osteosarcoma

Osteosarcoma is a malignant bone tumor characterized by the production of a malignant osteoid matrix by proliferating, dysregulated osteoblastic precursor cells. Patients typically present with progressive, localized bone pain and, less commonly, a palpable mass. Pathologic fractures may occur [20], whereas systemic symptoms are uncommon unless metastatic disease is present. A bimodal age distribution is recognized, with a primary peak in adolescents and young adults, and a second smaller peak in adults >60 years of age, often in the context of preexisting bone disease such as Paget disease [21]. The tumor is slightly more common in males [22,23].

Subtype recognition is clinically important because certain variants demonstrate distinctive imaging features. For example, osteoblastic osteosarcoma can, in some cases, exhibit an “ivory vertebra” appearance [24]; although there are other more common causes for this phenomenon. On radiographs, conventional osteosarcoma appears aggressive with mixed lytic and sclerotic change, bone destruction with a wide zone of transition, a permeative appearance, tumor ossification, and a soft tissue component. An aggressive periosteal reaction is common and may be visualized as sunburst, Codman triangle, or lamellated (onion skin) patterns.

**Figure 5:**
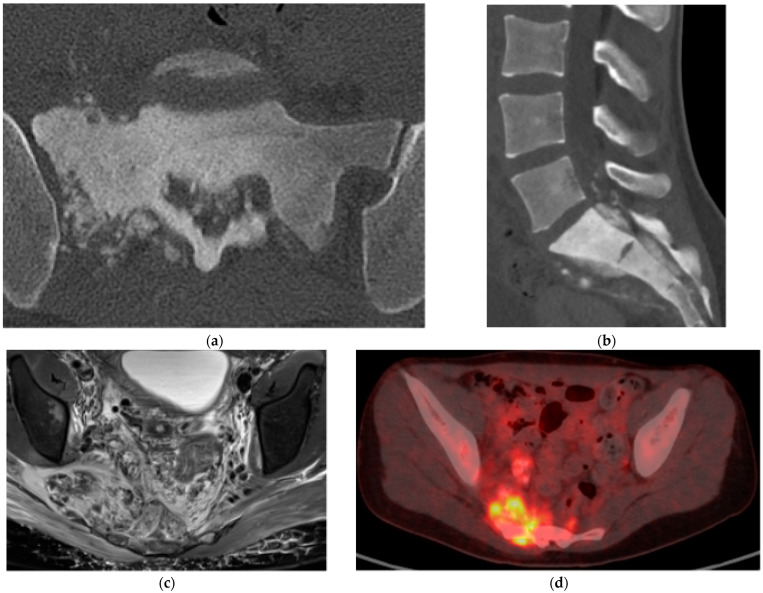
Osteosarcoma. (**a**) Axial CT bone window, aggressive, sclerotic lesion centered in the right sacral ala with new bone formation (osteoid matrix). (**b**) Sagittal CT bone window, osteoid matrix in the sacral canal and in the presacral space. (**c**) Axial T2 MR, large soft tissue mass extending into the pelvis. Multiple low-signal-intensity nodules compatible with osteoid matrix. (**d**) FDG PET/CT, increased uptake in the mass representing increased metabolic activity.

CT is the preferred imaging modality to demonstrate the osteoid matrix (Figure 5a,b) and facilitates both biopsy and surgical planning. MRI complements CT for local staging, assessing marrow and soft tissue invasion. T1 and T2 signal intensities are variable, reflecting the relative proportion of mineralized and nonmineralized tumor components; intralesional hemorrhage may also alter signal characteristics depending on the age of blood products. On MRI, telangiectatic osteosarcoma often demonstrates fluid–fluid levels with thick enhancing septa and nodular solid components, helping distinguish it from aneurysmal bone cyst, which lacks nodular enhancing soft tissue.

Post-gadolinium images show enhancement of the solid components (Figure 5c).

Osteosarcomas are typically avid on fluorodeoxyglucose (FDG) PET given their high metabolic activity (Figure 5d). PET can be useful in detecting metastatic disease and in monitoring treatment response. Dynamic contrast-enhanced MRI and diffusion-weighted imaging have utility in characterizing the tumor (e.g., vascularity) as well as response to treatment.

### 2.2. Chondrogenic

#### 2.2.1. Osteochondroma

Osteochondroma is the most common benign bone tumor, characterized by a cartilage-capped bony outgrowth that demonstrates cortical and medullary continuity with the parent bone [25]. Most are asymptomatic and discovered incidentally; symptomatic cases may present with pain, a palpable mass, or mechanical symptoms. There is a slight male predominance, and presentation is most common in the first two decades of life [26], although many lesions likely go undiagnosed.

**Figure 6:**
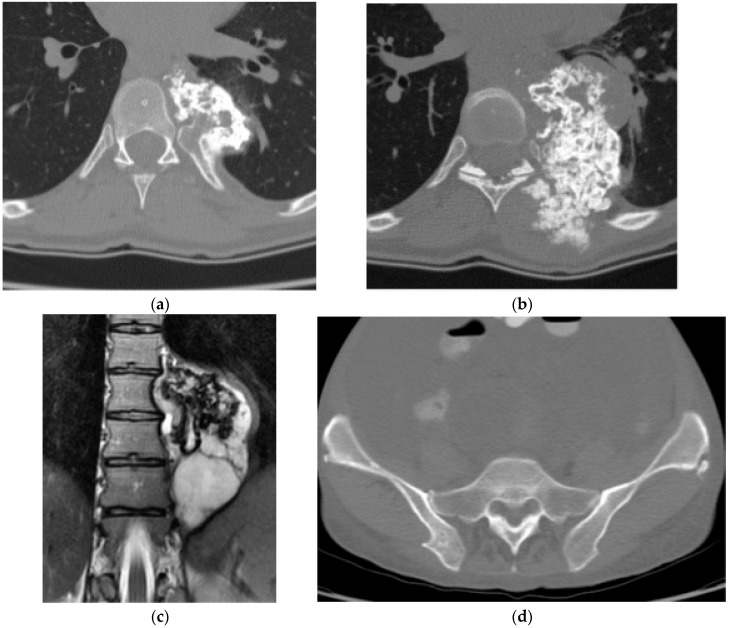
Osteochondroma with malignant, sarcomatous transformation. (**a**) Axial CT bone window, pedunculated osseous mass in a patient with multiple hereditary exostoses (MHE) with medullary cavity and cortex that is congruent with the normal adjacent bone. (**b**) Extensive calcifications are present. (**c**) Coronal T2 MR: low T2 signal indicates the calcification seen on CT. Large cystic component is present likely representing necrosis. (**d**) Small osteochondroma is present in the right iliac bone in this same patient with MHE.

**Figure 7:**
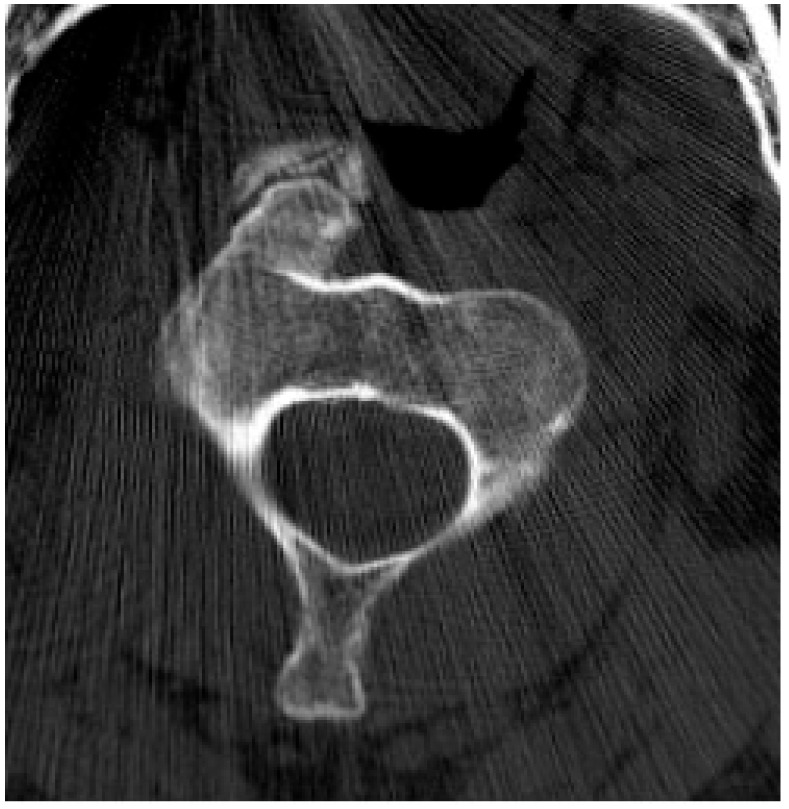
Osteochondroma. Axial CT bone window, lesion extending from the anterior aspect of the vertebral body with the medullary cavity and cortex congruent with the normal adjacent bone.

Radiographs and CT demonstrate a sessile or pedunculated osseous projection whose cortex and medullary cavity are continuous with those of the underlying bone (Figure 6a,d and Figure 7). In the spine, CT is preferred to radiography, as small lesions may be overlooked. However, evaluation of the cartilage cap is suboptimal on CT and radiographs, making MRI the modality of choice for comprehensive assessment.

On MRI, the osseous component of an osteochondroma follows signal intensity identical to that of normal adjacent bone (Figure 6c). The cartilage cap, like cartilage elsewhere in the body, is hypointense on T1- and hyperintense on T2-weighted images, although the signal can be variable depending on the degree of mineralization [27,28]. The cartilage cap is typically <1.5 cm thick. Post-contrast MRI may show thin peripheral enhancement of fibrovascular tissue, whereas the cartilage itself demonstrates minimal internal enhancement.

Both CT and MRI are useful for symptomatic lesions (including assessment for spinal canal or neural foraminal narrowing) and for detecting possible malignant transformation. The most common type of sarcomatous degeneration is into chondrosarcoma, which occurs in approximately 1–3% of solitary cases and 10–25% in patients with multiple hereditary exostoses (Figure 6) [28,29,30]. Transformation most often occurs in the pelvis and is rare in the spine [30]. Features worrisome for malignant transformation include new or increasing pain, and continued growth after skeletal maturity, with most transformation occurring after 20 years of age [27]. Radiographs or CT may show interval increase in size, areas of necrosis (often new lucency) [27], and development of an irregular soft tissue mass with calcifications (Figure 6a,b). Evaluation of the cartilaginous cap is best performed with MRI where measurement of the thickness can be made in detail. A cap thickness >1.5 cm is a sensitive but nonspecific finding for malignant transformation [27].

Overall, determination of malignant transformation is determined with biopsy; however, clinical and imaging findings are important in determining when to perform one.

#### 2.2.2. Chondroblastoma

Chondroblastoma is a rare, benign (but possibly locally aggressive) cartilage-forming tumor composed of proliferating chondroblasts, with admixed giant cells and chondroid matrix. It typically arises in the epiphysis of long bones, shows a strong male predominance, and peaks in the second decade of life [31,32,33]. Pain is common and may precede diagnosis by several months [32]. Imaging findings can appear aggressive across modalities but are nonspecific, and histopathologic confirmation is required.

**Figure 8:**
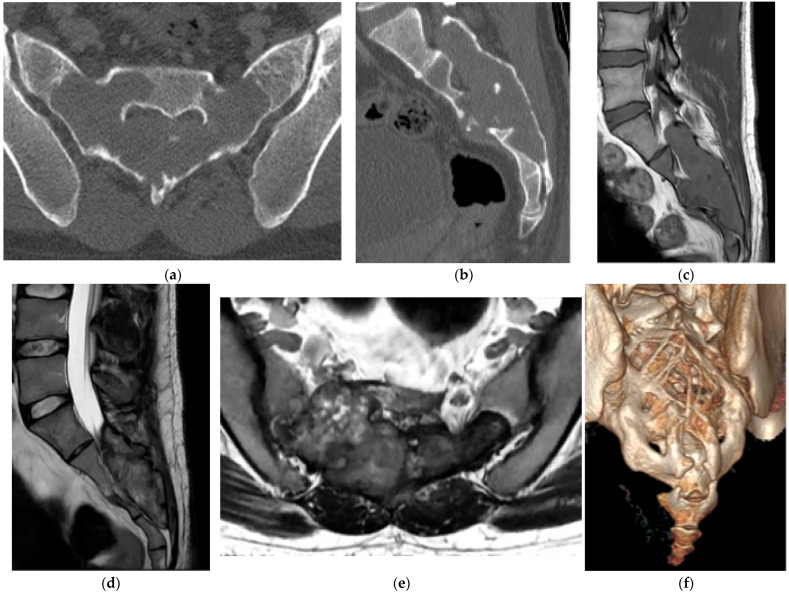
Chondroblastoma. (**a**) Axial and (**b**) sagittal CT bone window, expansile and lytic mass centered in the sacrum. No matrix calcification is present in this case. There is severe narrowing of the sacral canal. (**c**) Sagittal T1 MR: the mass is isointense to the paraspinal muscles. (**d**) Sagittal T2 MR: there is heterogeneously high T2 signal representing chondroid elements. (**e**) Axial T2 MR: the mass is expansile and causes severe narrowing of the sacral canal. (**f**) Volume-rendered reconstruction shows the severely thinned and remodeled cortex of the posterior sacrum.

Compared with appendicular lesions, vertebral chondroblastomas more often present with a large soft tissue mass with osseous destruction and spinal canal involvement (Figure 8). Rarely, extensive lesions have been reported to cause obstructive uropathy [34]. On radiographs and CT, chondroblastomas appear as expansile lytic lesions with sclerotic margins (Figure 8a,b). Matrix calcification is present in approximately 35–50% of cases and is best visualized on CT [35].

There is a close association with aneurysmal bone cysts, reported in up to one-third of chondroblastomas; therefore, assessment for the presence of cystic spaces with fluid–fluid levels should prompt consideration of an associated aneurysmal bone cyst (ABC), though pure ABCs lack solid enhancing tumor components [35].

On MRI, vertebral chondroblastomas usually demonstrate a large soft tissue mass with osseous destruction present. They are hypo- to isointense to muscle on T1 and heterogeneously hyperintense on T2 and STIR sequences (Figure 8c–e). Low T2/STIR signal regions correspond to internal calcifications, while higher T2 signal areas are thought to represent the chondroid elements [36]. Post-contrast MRI demonstrates enhancement of solid components. Prominent perilesional marrow and soft tissue edema may lead to overestimation of tumor size, similar to osteoblastoma [36].

Ultimately, given the overlap of imaging features, the differential diagnosis for these lesions includes chondrosarcoma, osteosarcoma, osteoblastoma, and metastasis. Histopathologic confirmation remains essential for diagnosis.

#### 2.2.3. Chondrosarcoma

Chondrosarcoma is a malignant, cartilage-producing bone tumor with a slight male predominance; most patients present in the fourth to seventh decades of life [37]. Presentation is typically insidious, with progressive dull/aching pain, and a palpable mass may be present [38]. Higher-grade lesions generally progress more rapidly and may lead to pathologic fracture [38,39].

**Figure 9:**
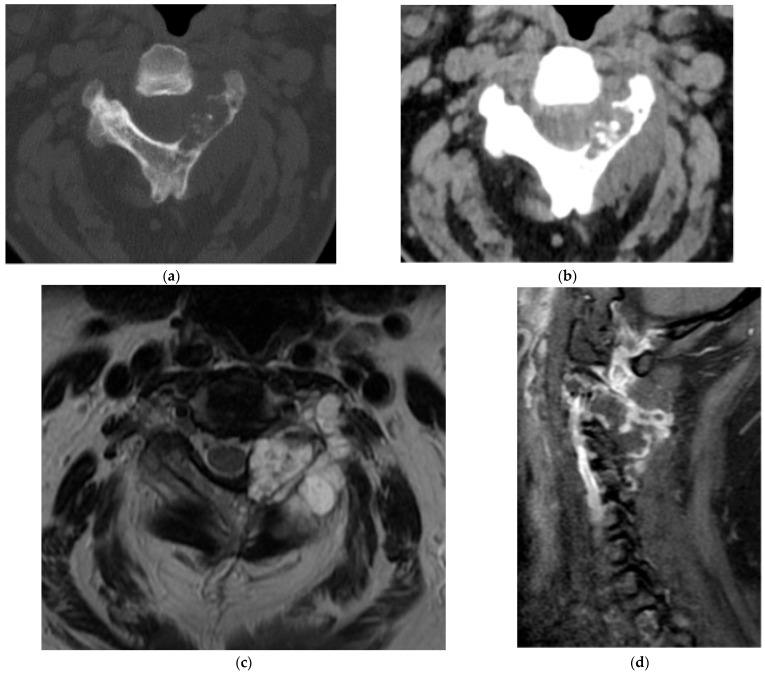
Chondrosarcoma. (**a**) Axial CT bone window, aggressive mass centered in the left lamina of C3 with chondroid matrix present centrally (rings and arcs). There is endosteal scalloping and cortical destruction. (**b**) Axial CT soft tissue window, extraosseous soft tissue component extending posteriorly from the left lamina mass. Chondroid matrix is again noted centrally. (**c**) Axial T2 MR: there is high T2 signal related to hyaline cartilage and increased water content. Mineralized “rings and arcs” are present centrally and low in signal. (**d**) Sagittal T1 fat saturation post-contrast, irregular peripheral enhancement.

Low-grade chondrosarcomas often appear lytic with chondroid matrix calcification, producing the characteristic “rings and arcs” appearance (Figure 9a,b). Endosteal scalloping, which is best visualized on CT (Figure 9a,b), is a hallmark feature, typically involving more than two-thirds of the cortex [40]. Higher-grade tumors may demonstrate a permeative appearance, cortical breach, and an associated paravertebral soft tissue component with heterogeneous enhancement. Contiguous vertebral involvement may also occur.

On MRI, chondrosarcomas demonstrate low to isointense T1 and high T2 signal intensity, reflecting the high water content of hyaline cartilage (Figure 9c) [41]. The mineralized “rings and arcs” are low signal on both T1- and T2-weighted images. Gradient echo or susceptibility weighted imaging, which are not commonly used in spine imaging, may show blooming of these mineralized portions. Post-gadolinium images demonstrate moderate to intense peripheral, septal, homogeneous, and/or nodular enhancement depending on the degree of septations and hyaline cartilage matrix (Figure 9d) [29]. Higher-grade lesions may not have septations and show more heterogenous enhancement [42].

Secondary chondrosarcomas account for approximately 12% of all chondrosarcomas [42]. Although spinal enchondromas are rare (~3% of all enchondromas), several features help distinguish them from low-grade chondrosarcomas [43]. Chondrosarcomas are typically larger (>5 cm), can demonstrate a cortical breach, and have endosteal scalloping [43]. Higher-grade lesions may exhibit a permeative appearance and/or a soft tissue component.

When radiographs or cross-sectional imaging are equivocal, bone scintigraphy can be supportive, as chondrosarcomas typically demonstrate increased uptake, whereas enchondromas often have low or absent activity [43]. Clinically, chondrosarcomas typically occur in an older population and almost always present with pain.

Clinical and imaging clues of the malignant transformation of an osteochondroma into a secondary chondrosarcoma include new or increasing pain, interval size increase, development of new surface irregularity (the most predictive radiographic sign), new cortical irregularities in the adjacent parent bone, and new areas of heterogeneous mineralization within the lesion [44]. Additional concerning findings include irregularity, thickening, or destruction of the cartilaginous cap, best visualized on MRI, which also aids evaluation of any new soft tissue component. Further, recent work on long bones suggest that dynamic contrast-enhanced MRI may help differentiate atypical cartilaginous tumors from high-grade sarcomas [45].

### 2.3. Notochordal

#### 2.3.1. Benign Notochordal Cell Tumors (BNCT)

BNCTs are intraosseous lesions of notochordal origin, more commonly encountered in the axial skeleton. They are typically incidental findings, with most patients asymptomatic, though mild pain can occur [46]. BNCTs are more frequently observed in adults and show no clear sex predilection.

**Figure 10:**
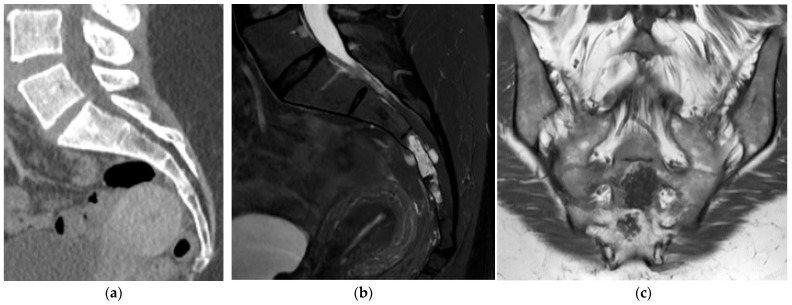
Benign Notochordal Cell Tumors. (**a**) Sagittal CT, very mildly increased sclerosis in the S3 body. (**b**) Sagittal STIR MR, hyperintense lesion in S3 without cortical expansion or soft tissue component. A second lesion of similar imaging characteristic is present in S4. The small cystic lesion in the adjacent sacral canal represents a perineural cyst. (**c**) Coronal oblique T1 MR: lesion demonstrates low T1 signal.

They are generally not visualized on radiographs. On CT, BNCTs appear as focal sclerosis with preserved bone architecture present (Figure 10a) [13], and without cortical destruction or soft tissue components (Figure 10a). These features help distinguish BNCTs from chordomas.

On MRI, BNCTs demonstrate similar signal to chordomas with low T1 and high T2/STIR signal intensity (Figure 10b,c). However, unlike chordomas, BNCT do not typically demonstrate convincing enhancement, which may aid initial radiologic assessment [47]; however, biopsy is still key in providing a definitive diagnosis.

#### 2.3.2. Chordoma

Chordoma is a rare malignant tumor arising from notochordal remnants, most commonly involving the axial skeleton—particularly the clivus, sacrum, and mobile spine [48,49]. Symptoms are location-dependent and include chronic and progressive pain, neurological deficits, and bowel or bladder dysfunction in sacral lesions [50]. Chordomas show a male predilection [51], with peak incidence in the sixth to seventh decades of life [49,52].

Radiographs may demonstrate an expansile lytic lesion with a large soft tissue component; however, CT and MRI are key in the evaluation. Chordomas are typically located in the midline, reflecting there notochordal origin, which can help to distinguish them from chondrosarcomas, which more often arise off midline. Clival chordomas may project posteriorly, and can indent the pons, producing a “thumb sign” appearance [53]. In the spine, chordomas may extend across the disc space and involve multiple levels.

**Figure 11:**
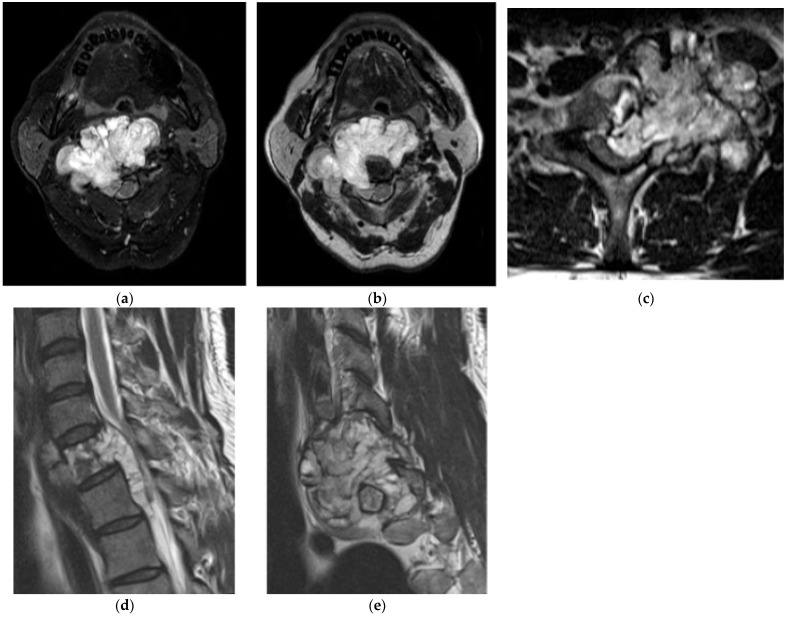
Chordoma. (**a**) Axial FS T2 MR and (**b**) axial T2 MR, expansile mass in a cervical spine vertebral body with high signal and interspersed low-signal areas indicating high fluid content and fibrous septa, respectively. (**c**) Axial T2 MR, chordoma in the C7 vertebral body with characteristic high signal on T2 with low-signal fibrous septa. (**d**) Sagittal T2 MR, high signal intensity on T2 with multiple fibrous septa. (**e**) Sagittal T2 MR: midline extension of the mass into the spinal canal results in severe canal narrowing and cord compression.

On CT, they are typically uniform attenuation unless hemorrhage or necrosis is present. There may be sparse internal calcifications which are thought to represent foci of normal residual bone. On MRI, the T1 signal can be variable depending on the extent of intralesional mucus and hemorrhage [29]. T2-weighted images classically show high signal intensity secondary to high fluid content with areas of hypointensity representing interspersed fibrous septa (Figure 11) [13,54]. Heterogeneous and sometimes “ring and arc” enhancement is seen after contrast administration [13]. Bone scintigraphy is of limited value due to variable uptake, and angiography is generally not helpful, as chordomas are not markedly hypervascular.

### 2.4. Hematolymphoid

#### 2.4.1. Plasmacytoma

Spinal plasmacytoma is a localized proliferation of monoclonal plasma cells within bone. Presentation is typically insidious, most often with progressive, localized pain; neurological deficits can occur with spinal cord or nerve-root compression. Unlike multiple myeloma, systemic symptoms are usually absent. This condition most commonly affects males over the age of 50 [55].

**Figure 12:**
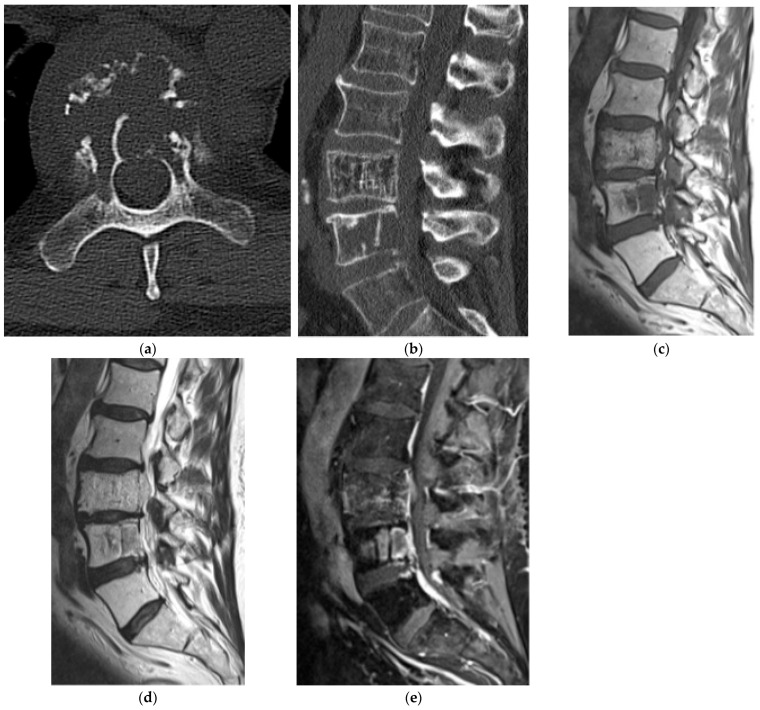
Plasmacytoma. (**a**) Axial CT bone window, thickened trabeculae with cortical destruction in the L5 vertebral body; this is the characteristic “mini brain” (cerebriform) appearance. (**b**) Sagittal CT bone window, L4 plasmacytoma with thickened residual trabeculae. Notice a hemangioma in the L3 vertebral body which has thin trabeculae (corduroy sign). (**c**,**d**) Sagittal T1 and T2 MR, L4 plasmacytoma with low T1 and T2 signal. (**e**) Sagittal T1 fat saturation post-contrast, relatively homogeneous enhancement in the plasmacytoma with ventral epidural enhancement possibly representing a combination of epidural disease or venous congestion.

**Figure 13:**
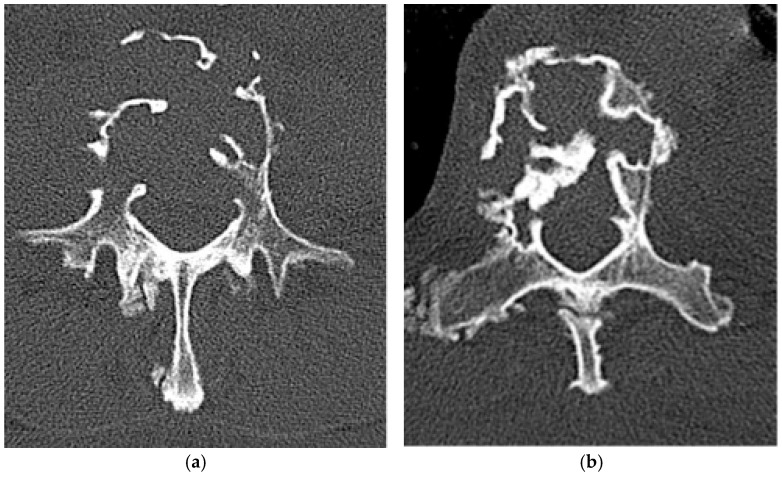
Plasmacytoma. (**a**,**b**) Axial CTs bone window. Plasmacytomas in two different patients demonstrate the characteristic “mini-brain” (i.e., cerebriform) appearance with thickening of the intact trabeculations.

On radiographs and CT, lesions are typically large, expansile, and lytic, with an associated soft tissue mass; cortical thinning and destruction may be present (Figure 12a,b and Figure 13). Solitary spinal plasmacytomas may show thickened cortical bony struts which are thought to represent residual bone. This residual bone is postulated to be thickened due to the increased stress applied on it as lysis of the normal bone occurs [56]. On CT and MRI, this phenomenon gives the characteristic “mini-brain” (i.e., cerebriform) appearance, which strongly suggests the diagnosis (Figure 12a,b and Figure 13) [56].

MRI internal signal is variable and therefore less diagnostic, with heterogenous T1 and T2 signal and variable enhancement (Figure 12c–e). Solitary bone plasmacytoma carries a higher risk of progression to multiple myeloma than extramedullary plasmacytoma [57], making surveillance essential. Serum-free light chains are monitored for progression, and whole body MRI is a prognostically relevant examination to assess for additional lesions that would upstage the disease to myeloma (International Myeloma Working Group) [57]. If whole body MRI is unavailable, PET/CT is an acceptable alternative.

#### 2.4.2. Lymphoma

Primary lymphoma of the osseous spine is a rare extranodal malignancy, most commonly of the diffuse large-B-cell subtype [58]. Patients often present with localized bone pain, a palpable mass, and, in advanced cases, pathological fractures may occur. Neurologic complications (e.g., radiculopathy, myelopathy, or cauda equina syndrome) depend on the level and extent of spine involvement. Systemic “B” symptoms are less common than in nodal lymphomas [58,59]. The disease shows a slight male predominance and most frequently affects middle- to older-aged adults [58,59].

Lymphoma exhibits variable radiographic appearances, most often osteolytic, but occasionally osteosclerotic or mixed, the latter more typical of Hodgkin lymphoma [13]. The classic lytic pattern demonstrates permeative or moth-eaten destruction with a wide zone of transition [60]. Although sclerotic lesions are less common, they may arise post treatment of lytic disease. This sclerotic disease may involve the entire vertebral body diffusely in which case the term “ivory vertebrae” can be used. Vertebra plana (complete collapse of the vertebral body) can occur but is nonspecific and may also be seen in children with Langerhans cell histiocytosis.

**Figure 14:**
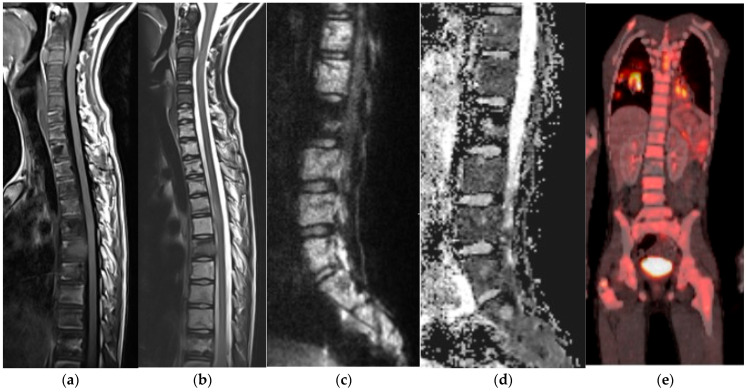
Lymphoma. (**a**) Sagittal T1 and (**b**) sagittal T2 MR, extensive marrow infiltrate with loss of normal fatty marrow signal on T1 and T2 sequences. (**c**) B-1000 and (**d**) ADC map MR sequences: restricted diffusion in the involved marrow helps diagnose lymphoma. (**e**) FDG PET/CT, tracer uptake in the bone marrow and right lung; PET helps with staging of lymphoma.

Compared with radiographs, CT more clearly demonstrates extraosseous extension, periosteal reaction, and osseous destruction. MRI is the most sensitive modality for early marrow infiltration, with low T1 signal reflecting fatty marrow replacement (Figure 14a). T2-weighted images are variable, most frequently with hyperintense signal, but may be low signal when fibrosis is present (Figure 14b). Similar to lymphoma elsewhere in the body, enhancement is typically homogeneous and diffuse. Lymphoma has increased signal on diffusion-weighted images (i.e., restricted diffusion) due to increased cellularity with a high nucleus-to-cytoplasm ratio (Figure 14c,d).

Osseous lesions are the most common, followed by spinal canal deposits, which may be extradural, intradural extramedullary, or intradural intramedullary [61]. These deposits are best evaluated with MRI and are more often seen in disseminated disease. PET/CT helps to assess for metastatic deposits, which is integral for staging and prognosis (Figure 14e). Lymphoma is avid on FDG PET, with uptake decreasing following successful treatment. Given its hypermetabolic nature, lymphoma may also show increased Thallium-201 uptake, which functions as a potassium analog taken up by active tumor cells.

### 2.5. Vascular/Cystic

#### 2.5.1. Hemangioma

Vertebral hemangiomas (VH) are benign vascular lesions composed of thin-walled proliferating vessels with variable fat content. VH are common in the general population, typically asymptomatic, increase in prevalence with age, and show no clear sex predilection [62,63]. Histologically, hemangiomas are composed of dilated vascular channels within a fatty stroma, insinuating between vertically oriented osseous trabecula, sometimes remodeling although not destroying the bone [29]. Accumulation of this adipose tissue is secondary to reactive changes causing involution of normal marrow [64]. This architecture explains their characteristic imaging appearance, although atypical hemangiomas are lipid poor.

**Figure 15:**
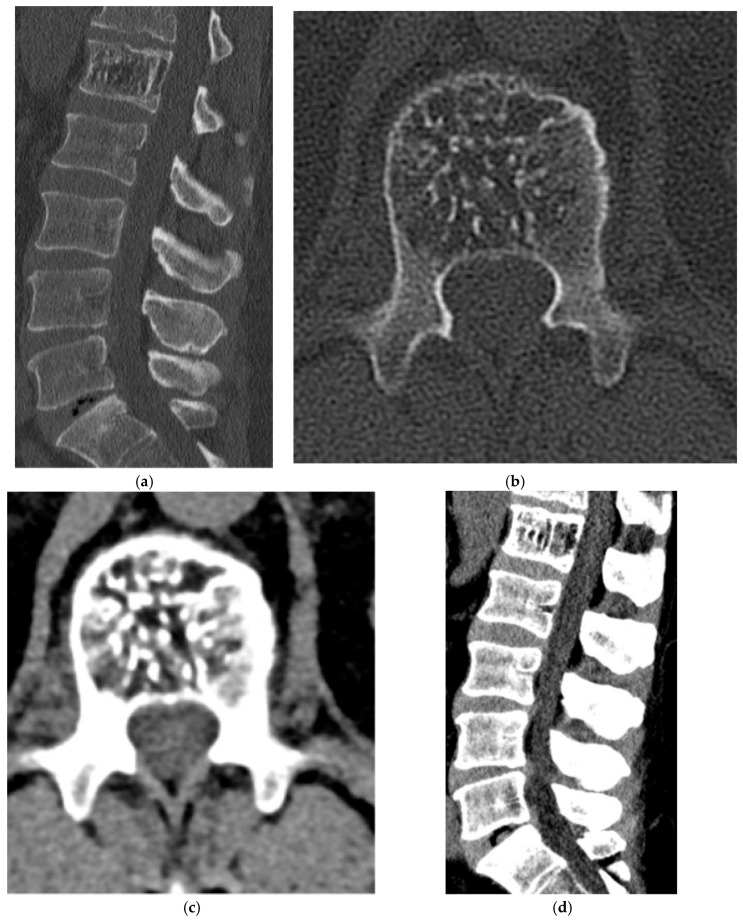
Hemangioma. (**a**) Sagittal CT bone window, hemangioma in the L1 vertebral body with the characteristic “corduroy” appearance. (**b**) Axial CT bone window, classic “polka dot” appearance of a hemangioma with the dots representing intact trabeculae. (**c**) Axial and (**d**) sagittal CT soft tissue window: intralesional fat is typical in hemangiomas.

Radiographs demonstrate distinct trabeculations within a vertebral body of lower density than the adjacent segments. Correspondingly, CT demonstrates a low-density lesion with prominent, vertically oriented trabeculations, which give rise to the classic “polka dot” sign on axial sections and the “corduroy” appearance on sagittal or coronal sections (Figure 15a,b). Intralesional fat may be visualized with CT (Figure 15c,d).

**Figure 16:**
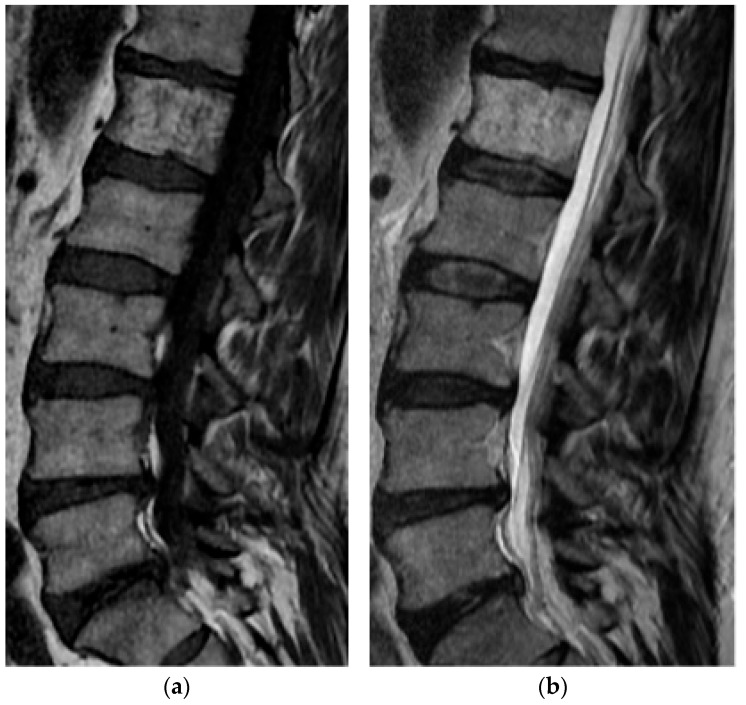
Hemangioma. (**a**) Sagittal MR T1- and (**b**) sagittal MR T2-weighted images: the L1 vertebral body hemangioma has increased signal on T1 and T2 secondary to intralesional fat and fluid, respectively. Vertical striations are in keeping with thinned but intact trabeculae.

On MRI, typical hemangiomas are T1 and T2 hyperintense, reflecting fat and vascular content, respectively (Figure 16a,b). On fat saturation techniques, there is a drop-out of the signal, in keeping with macroscopic fat. On post-contrast MRI, these lesions typically enhance avidly. Given their decreased fat content, atypical hemangiomas may be dark on T1, although they also enhance quite avidly, a feature that may mimic metastases. In such cases, CT may be of greater diagnostic value, as trabeculations are usually present and may be more readily identified. In equivocal cases of hemangiomas versus metastases, preservation of the characteristic trabecular pattern and overall bone remodeling rather than frank destruction favors VH over metastasis. Nuclear medicine uptake is variable and therefore not diagnostic [13]. Catheter angiography demonstrates hypervascularity, consistent with the underlying pathology.

**Figure 17:**
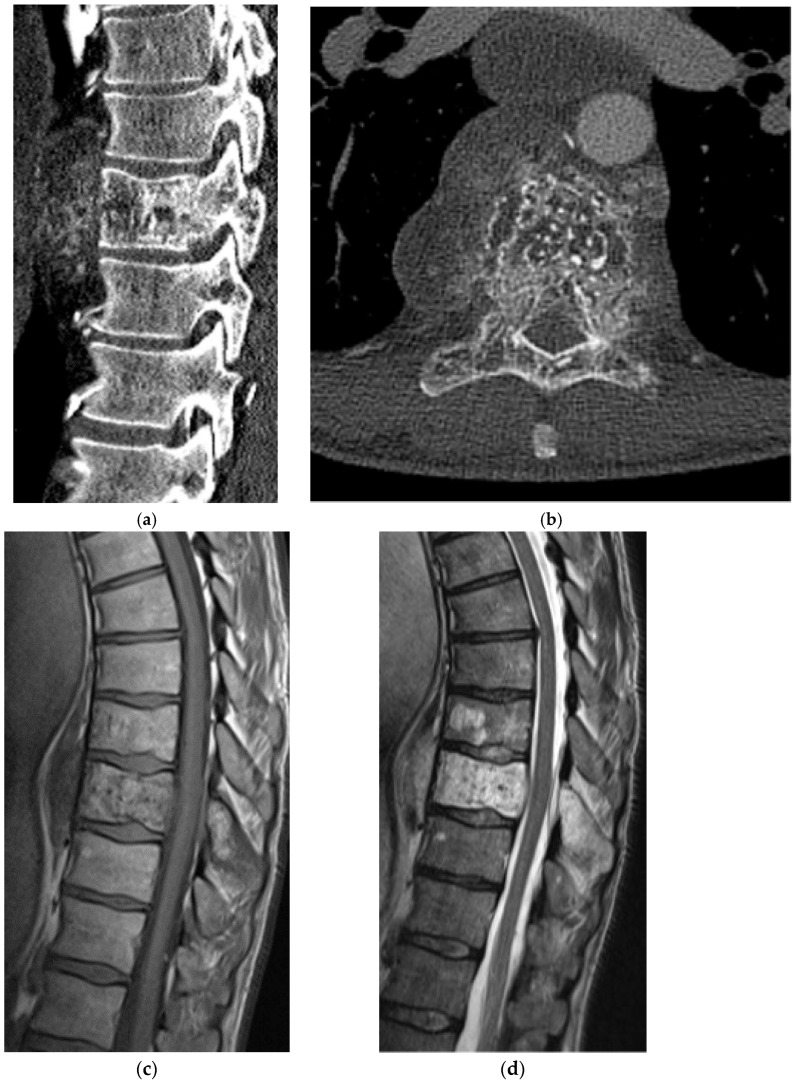

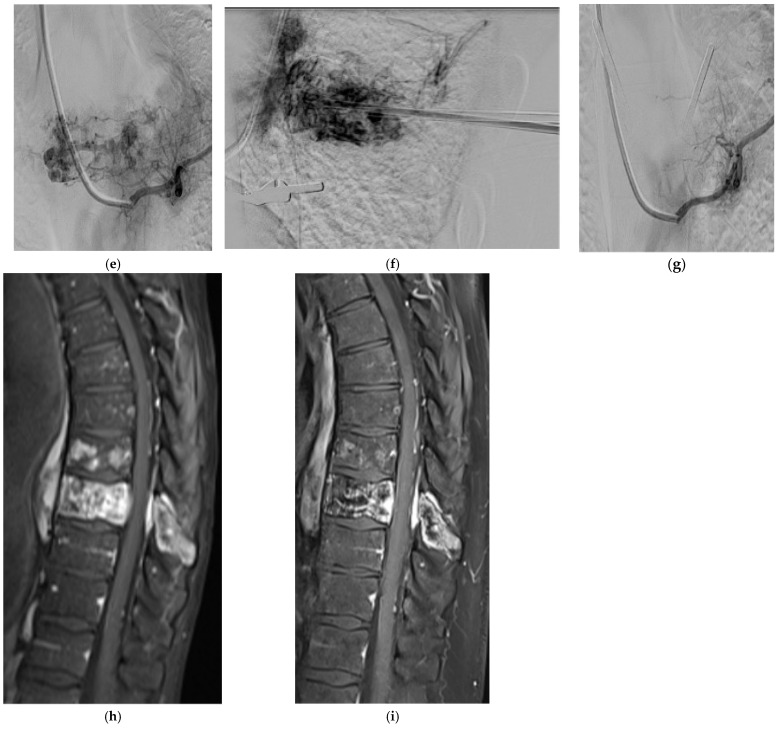
Aggressive hemangioma. (**a**) Sagittal CT, osseous expansion, and extensive extraosseous extension into the paravertebral soft tissues. (**b**) Axial CT: osseous expansion results in narrowing of the spinal canal. (**c**) Sagittal T1 MR: the low signal is unlike that of a typical, nonaggressive hemangioma. (**d**) Sagittal T2 MR: there is osseous expansion with outward bowing of the posterior cortex of the involved vertebral body. Extraosseous extension is visualized in the prevertebral space. (**e**) Catheter angiogram with injection in the segmental artery demonstrates prominent vascular channels in the aggressive hemangioma. (**f**) Fluoroscopic images during liquid embolization of the lesion through a transpedicular approach. (**g**) Post-procedural catheter angiogram demonstrates marked reduction in vascularity in keeping with successful treatment. (**h**) Pre-procedural T1 FS contrast-enhanced MR demonstrates the avid enhancement associated with the aggressive hemangioma. (**i**) Post-procedural follow-up T1 FS contrast-enhanced MR shows marked reduction in enhancement in keeping with interval embolization.

Aggressive hemangiomas can demonstrate extensive extraosseous extension into adjacent soft tissue (Figure 17a,b) [65], neural foramina, and/or the spinal canal, potentially causing neurologic compromise (Figure 17b) [64]. On MRI, in contrast to typical hemangiomas, there may be low T1 signal, particularly in the extraosseous components, although they maintain their T2 hyperintensity (Figure 17c) and avid contrast enhancement (Figure 17d) [64]. Catheter angiography typically reveals extensive vascularity, with progressive opacification of intraosseous followed by extraosseous components (Figure 17e–g). Management of these lesions can be complex, and preoperative embolization is often performed to reduce intraoperative blood loss prior to surgical intervention (Figure 17e–i).

#### 2.5.2. Aneurysmal Bone Cyst

Primary aneurysmal bone cysts (ABCs) of the spine are benign but often locally aggressive lesions composed of blood-filled cystic spaces lined by fibroblasts, reactive bone, and multinucleated giant cells. Patients typically present with localized pain and swelling, and neurological deficits may occur depending on spinal level and lesion size. ABCs show no clear sex predilection and most commonly affect children and young adults, though they can occur at any age [66,67].

**Figure 18:**
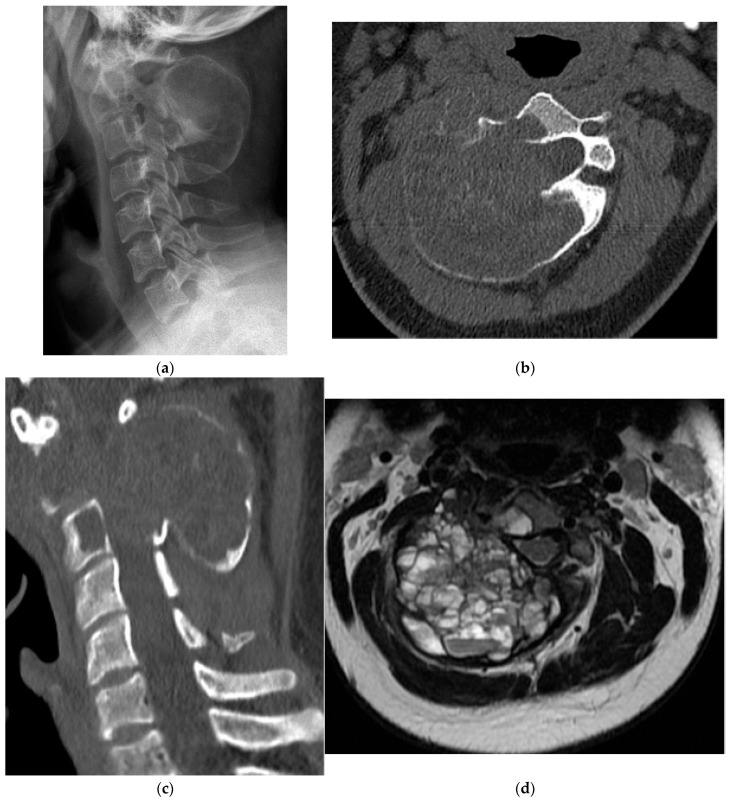
Aneurysmal bone cyst. (**a**) Large lucent expansile mass centered in the posterior elements of C2 and C3. (**b**) Large expansile mass in the posterior element of the C3 vertebral body. There is thinning of the cortex. (**c**) Sagittal CT bone window: the mass involves the posterior elements and the vertebral bodies of C2 and C3. (**d**) Axial T2 MR, multiple fluid–fluid levels representing serum hematocrit levels of intralesional blood.

On radiographs, ABCs appear as well-defined cystic lesions with variable-sized thin wall cavities, producing the classic “soap bubble” appearance (Figure 18a). The lesion is usually expansile but exhibits a narrow zone of transition, consistent with its benign nature. CT mirrors these findings, showing an expansile, multiloculated lesion which may extend into the adjacent vertebrae or soft tissues (Figure 18b,c). Fluid–fluid levels may be visible on CT but are more readily characterized on MRI, where they represent layering of blood products of differing ages due to hemorrhage and sedimentation of serum and cellular material.

On MRI, the internal cystic spaces demonstrate variable signal intensity depending on the age and composition of blood products (Figure 18d). The periphery may be low signal on T1 and T2 sequences reflecting thickened periosteum (Figure 18d). Contrast-enhanced CT and MRI typically show smooth enhancement of internal septations [13]. The presence of thick, irregular septations or enhancing nodular components should raise the suspicion for an underlying aggressive lesion, such as telangiectatic osteosarcoma, which can also demonstrate fluid–fluid levels.

Secondary ABCs may arise in the presence of other neoplasms, for example, osteosarcoma. In these cases, the malignant component is often smaller than the cystic component, making radiologic differentiation difficult; and poorly defined sclerosis may be present.

Bone scintigraphy may show peripheral uptake and central photopenia giving the appearance of a “doughnut sign,” however, this finding is not specific [68]. ABCs are typically hypervascular lesions; therefore, while angiography is not routinely required for diagnosis, it can play an important role in pre-procedural planning, particularly when embolization is considered, either as part of a preoperative resection strategy, or for primary endovascular management.

### 2.6. Fibro-Osseous

#### Fibrous Dysplasia

Spinal involvement in fibrous dysplasia (FD) is uncommon, representing fewer than 5% of all FD cases, and is usually part of a polyostotic pattern, given that single, monostotic spinal lesions are rare [69]. In FD, normal lamellar bone is replaced with irregular woven bone and fibrous tissue. Patients may present with localized pain, and neurological symptoms can arise from mass effect or pathologic fracture [70]. FD is most often diagnosed in adolescents and young adults.

**Figure 19:**
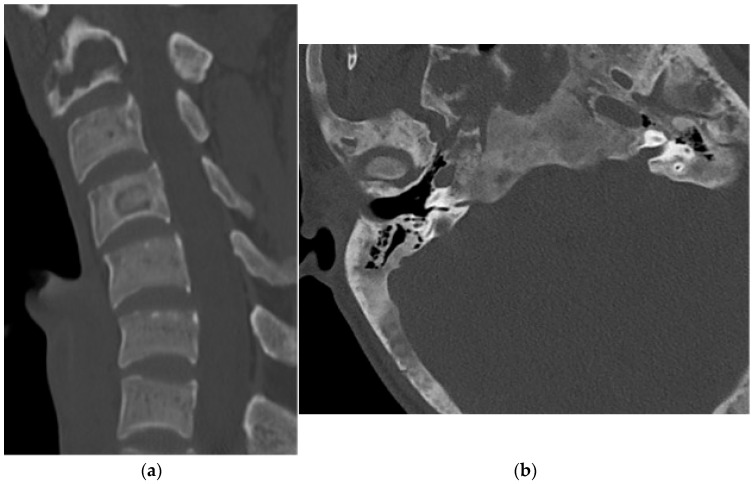
Fibrous dysplasia. (**a**) Sagittal CT bone window, “ground glass” lesion with peripheral lucency in the center of the C4 vertebral body. A lytic lesion with peripheral sclerosis is partially included in the C2 vertebral body. (**b**) Axial CT bone window through the skull base, extensive ground glass, and expansion in the calvarium at the skull base in a patient with McCune Albright syndrome.

The imaging features of spinal FD mirror those of FD elsewhere. Radiographic findings are variable and may show sclerotic, “ground glass,” or expansile lesions with endosteal scalloping, or a combination thereof. On CT, FD typically appears as an expansile vertebral lesion with ground glass opacities, nonaggressive lytic areas, and/or a sclerotic peripheral rim (Figure 19a,b). The cortex is usually intact but thinned from osseous remodeling and expansion. A soft tissue component may be present but does not necessarily indicate malignancy [69]. Extensive vertebral involvement may result in pathologic collapse, with potential canal or foraminal narrowing from fracture fragments or bulky fibro-osseous tissue.

Although fibrous tissue is typically T1 and T2 hypointense, MRI findings for FD are nonspecific and show variable signal on T1- and T2-weighted sequences [71], with enhancement generally ranging from moderate to avid. A “rind sign” has been described on MRI and the term is used when dense, sclerotic reactive bone is visualized at the lesion periphery [71]. Given these features, spinal FD can mimic more aggressive processes.

On bone scintigraphy, lesions typically show increased radiotracer uptake, which diminishes as these lesions become quiescent later into adulthood. Sarcomatous transformation is rare (<1% of cases) [72] and most often to osteosarcoma, though chondrosarcoma and undifferentiated pleomorphic sarcoma have also been reported [72].

### 2.7. Osteoclast-Rich Stromal

#### Giant Cell Tumor

Giant cell tumor (GCT) of the spine is rare and histologically benign but may exhibit locally aggressive behavior. Patients can present with localized pain, neurological deficits, and occasionally pathological fractures. GCTs most commonly involve the sacrum, show a slight female predominance, and are typically diagnosed in young adults [73].

**Figure 20:**
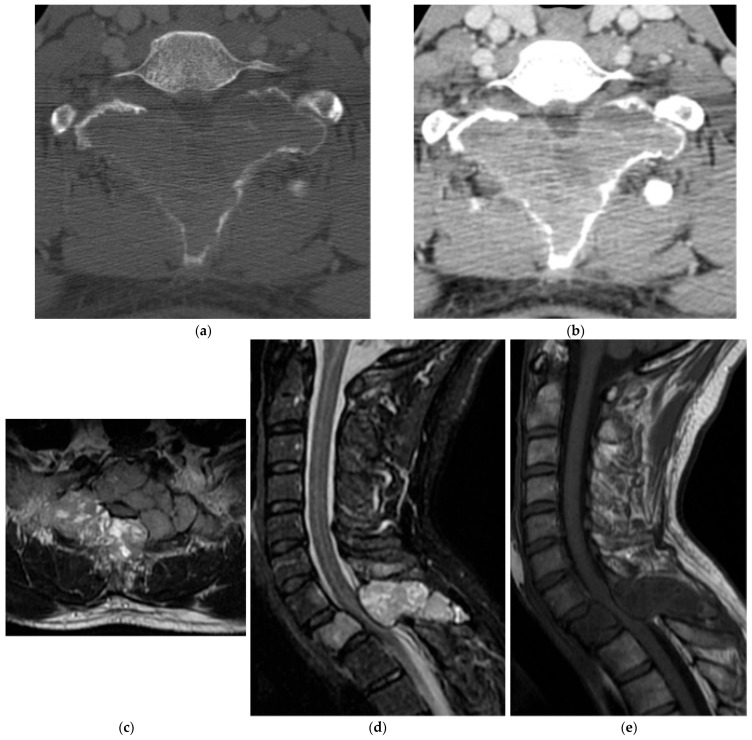
Giant cell tumor. (**a**) Axial CT bone window and (**b**) axial CT soft tissue window demonstrate a large, expansile mass in the posterior elements on the cervical spine. There is osseous remodeling with thinning of the cortex. This is a biopsy-proven giant cell tumor. (**c**) Axial T2 MRI shows an expansile mass with heterogeneously high-signal and low-signal septations in keeping with fibrous components. (**d**) Sagittal T2 MR shows the same giant cell tumor which demonstrates heterogeneously high signal with involvement of the vertebral body and posterior elements. There is severe spinal canal narrowing with compression of the cord. (**e**) Sagittal T1 MR sequence: giant cell tumor is typically intermediate to low signal on T1 as in this case.

**Figure 21:**
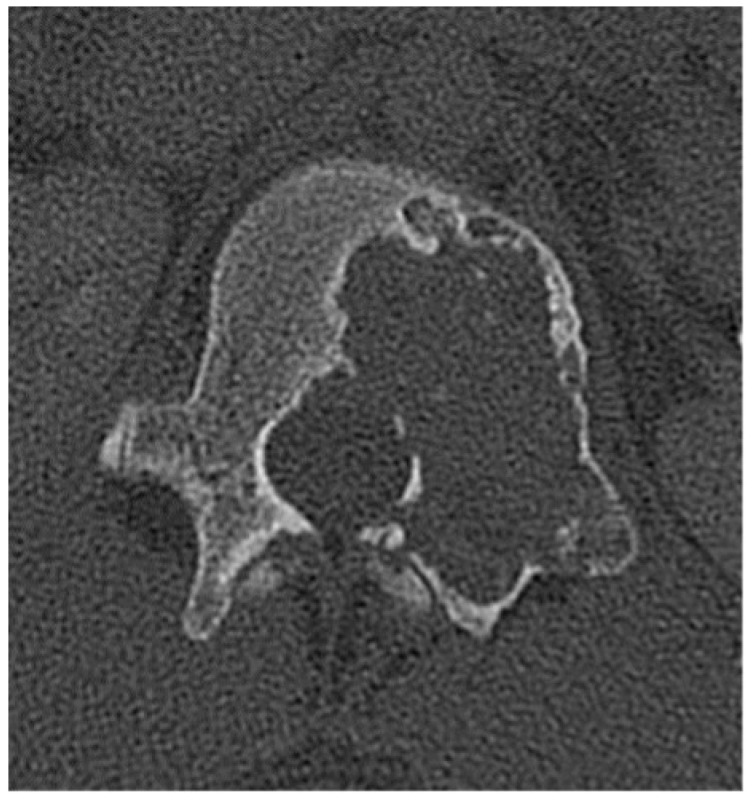
Giant cell tumor. Axial CT bone window demonstrates a giant cell tumor with minimal sclerosis present along the “non-eccentric” border of the lesion which has been described in the literature [74]. Small internally directed bony septations are present.

On radiographs, GCT appears as an expansile lytic lesion that thins and remodels the cortex, with a narrow zone of transition and little or no peripheral sclerosis. If lesions are large, pathologic fracture may occur. A soft tissue component may be seen on radiographs but is better evaluated with CT and MRI. CT is optimal for demonstrating cortical thinning or destruction and any soft tissue component (Figure 20a,b). Internal architecture can vary with intralesional hemorrhage or necrosis [74]. Interestingly, with eccentric lesions, the eccentric margin of the tumor may show cortical thinning/destruction, whereas the opposite, non-eccentric border may be sclerotic (Figure 21) [74]. Fine internal septations can occur but are nonspecific, as similar septations may be seen in hemangioma or plasmacytoma [74]. Notably, GCTs lack internal matrix mineralization.

On MRI, signal characteristics are variable, reflecting increased collagen content, fibrous components, and hemosiderin (Figure 20c) [75]. These lesions are predominantly low to intermediate T1 signal and heterogeneously high T2 signal, with enhancement of solid components (Figure 20d,e) [75]. Fluid–fluid levels may indicate an associated ABC.

Bone scintigraphy may be useful as GCTs often shows peripheral radiotracer uptake with central photopenia secondary to osteolysis and necrosis. This “doughnut sign” can also be seen with ABCs and is not specific for GCT.

### 2.8. Adipocytic

#### Lipoma

Primary spinal lipoma is a rare, benign neoplasm composed of mature adipocytes and may be intradural, extradural, or intraosseous. In children, spinal lipomas are often associated with spinal dysraphism and typically occur in the lumbosacral region [76]. Non-dysraphic lipomas are less common and usually present in adults [77,78]. Symptoms reflect size and location: mass effect or spinal cord tethering can cause pain, neurological deficits, and bladder/bowel dysfunction in lower spinal segments [76].

Milgram described three histopathologic involutional changes that occur within intraosseous lipomas and correlate with imaging characteristics [79]. A stage 1 lesion consists of adipocytes without abnormal cytologic features. In stage 2, fat necrosis and dystrophic calcifications start to appear. In stage 3, fat necrosis and calcifications predominate in addition to cyst formation and reactive ossification with occasional viable adipocytes present.

**Figure 22:**
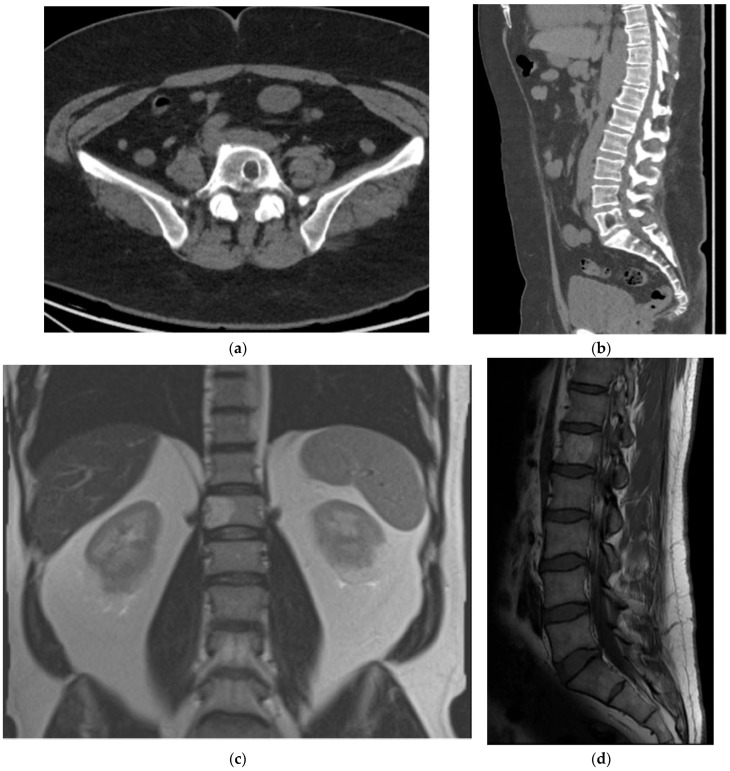
Lipoma. (**a**) Axial CT soft tissue window and (**b**) sagittal CT soft tissue window demonstrate a well-circumscribed, non-expansile, fat-attenuating lesion within the L5 vertebral body. The lesion is consistent with intraosseous lipoma. (**c**) Coronal T2 MR image shows a non-expansile intraosseous lesion confined within the vertebral body, with similar signal intensity to fat, in keeping with intraosseous lipoma. (**d**) Sagittal T1 MR (fast spin echo sequence) demonstrates the same intraosseous lipoma as in Figure 22c.

On radiographs, intraosseous lipomas can be subtle, but typically appear as well-defined, lucent lesions, which may be mildly expansile. On CT and MRI, these lesions follow fat attenuation and fat signal, respectively (Figure 22). Hemangiomas may mimic the appearance but can be differentiated based on their characteristic “polka dot” or “corduroy” appearance on CT and the presence of enhancement on MRI, which lipomas lack. The normal adjacent marrow signal will typically be lower than that of the lipoma.

Stage 2 and 3 lesions may show calcification (low signal on T1 and T2), and variable signal related to necrosis. On radiographs, stage 3 lesions often appear involuted and show a peripheral predominant pattern of ossification and variable presence of central ossification [79]. CT and MRI features become more variable with increasing involution; however, adipocytes remain and the recognition of this intralesional fat is a key diagnostic feature.

### 2.9. Bone Remodeling Disorder (Tumor-like)

#### Paget’s Disease

Paget disease of bone is a focal disorder of disorganized remodeling, driven by excessive osteoclastic resorption followed by excessive and abnormal osteoblastic activity. Patients are often asymptomatic, with detection occurring incidentally on imaging or through elevated serum alkaline phosphatase levels. When symptomatic, presentation may include mechanical pain or neurological deficits from bony overgrowth, spinal stenosis, nerve compression, or vertebral collapse [80,81]. Rarely, vascular steal phenomena may occur [80,81]. Affected individuals are also at increased risk for pathologic fracture and secondary osteoarthritis in adjacent joints [80]. Incidence rises with age (particularly >50 years) and shows a male predominance [82].

**Figure 23:**
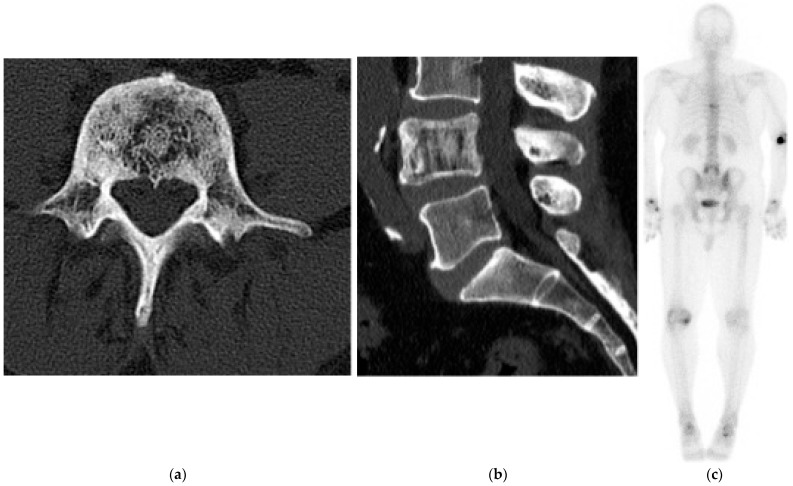
Paget. (**a**) Axial CT bone window, heterogenous appearance of the vertebral body and posterior elements with areas of lucency and areas of sclerosis with thickening of the trabeculae. (**b**) Sagittal CT bone window, thickening of the peripheral cortical bone and the central trabeculae. (**c**) MDP bone scan, corresponding increased uptake in the L4 vertebral body.

Imaging is considered an essential part of diagnosing Paget disease and reflects the stage of disease. In the early, destructive/lytic phase where osteoclasts predominate, radiographs will show areas of osteolysis and vertebral body expansion in the anterior–posterior and lateral planes without any loss in vertebral body height unless there is an underlying pathologic fracture [83]. A “ghost vertebra” appearance may be present (where the vertebra is not visualized) accounting for the lytic change and expansion [81]. CT may be used to confirm this lytic phase and differentiate it from other causes of osteolysis by better showing the expansion and trabecular hypertrophy that may not be as apparent on radiographs. This vertebral body expansion leads to squaring of the vertebral body which is seen as flattening of the normal concavity of the anterior margin [83]. The most commonly encountered stage radiographically is the mixed density phase where there is a combination of osteoclastic and osteoblastic activity [81]. In this phase, vertebral body involvement demonstrates thickened and hypertrophied trabeculae running parallel to the endplates as well as thickening of the peripheral cortical bone (Figure 23a,b). This osseous thickening/sclerosis leads to the characteristic imaging feature described as the “picture frame” sign which is considered pathognomonic for Paget disease [81]. Both radiographs and CT may demonstrate this finding. The late sclerotic phase, which is dominated by osteoblastic activity, leads to the finding of an “ivory vertebra,” characterized by increased density through the vertebral body [81].

MRI appearance also depends on the stage of disease, with variable T1 and T2 signal depending on the degree of trabecular and cortical thickening. The marrow cavity in the vertebral body may be reduced in size and/or replaced due to the thickening [81]. Bone scintigraphy is highly sensitive, showing early changes of increased blood flow and osteoblastic activity (Figure 23c) [83]. Scintigraphy may be used to determine whether there is active disease and to evaluate the overall extent and distribution.

### 2.10. Primitive Small Round Cell

#### Ewing Sarcoma

Ewing sarcoma is an aggressive, malignant small round cell tumor; spinal involvement is uncommon, accounting for approximately 10–15% of primary cases [84]. Most tumors harbor the t(11;22)(q24;q12) EWSR1-FLI1 fusion [84]. Ewing sarcoma predominantly affects children and young adults and patients often presents with nonspecific localized back pain; neurological deficits or, rarely, a palpable mass may occur [85]. Systemic symptoms are unusual and often signify advanced disease.

**Figure 24:**
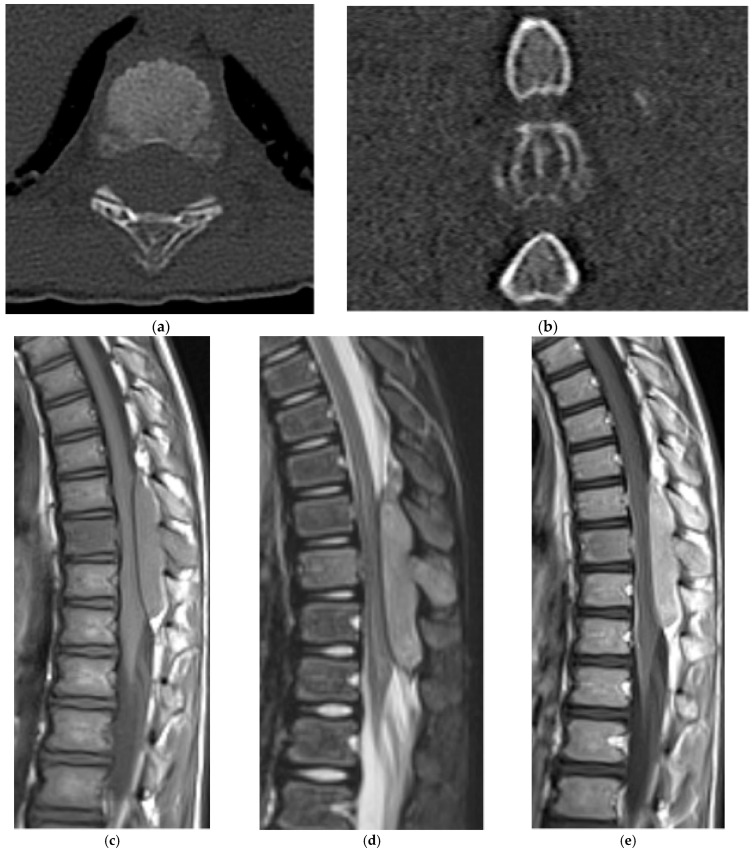
Ewing’s sarcoma. (**a**,**b**) Axial and coronal CT bone window, expansile lesion in the posterior elements of a lower thoracic vertebra with characteristic “onion skin” periosteal reaction. There is sclerosis of the vertebral body. (**c**) Sagittal T1 MR, intermediate signal intensity lesion involving the vertebral body with extension of the soft tissue component into the dorsal epidural space. (**d**) Sagittal T2 MR: lesion is mildly hyperintense. There is severe spinal canal narrowing with cord compression. (**e**) Sagittal T1 post-contrast MR, mildly increased enhancement.

Radiographs typically demonstrate a large, aggressive, lytic lesion with a wide zone of transition and a permeative and/or mottled pattern; vertebral height loss ranges from minimal to vertebra plana [86]. Periosteal reactions can be lamellated “onion skin,” Codman triangle, or “sunburst” patterns, like those seen in osteosarcoma. Of these, the lamellated “onion skin” appearance is most associated with Ewing sarcoma, although it is rarer in the spine (Figure 24a,b) [86], and not specific as it can be seen with osteomyelitis [86]. Unlike osteosarcomas, new bone formation is uncommon. Soft tissue extension is typical and well visualized on CT and MRI (Figure 24c–e). The soft tissue component in Ewing sarcoma is usually larger than the osseous component (Figure 24c–e) [29]. CT and MRI help to evaluate the extent of soft tissue extension and the relation between the mass and important neurologic structures (Figure 24c–e).

On MRI, lesions are iso- to hypointense on T1 and hyperintense on T2, with heterogeneous post-contrast enhancement and restricted diffusion reflecting high cellularity. Rarely, marked necrosis and reactive bone formation in the osseous component of the mass may produce a purely sclerotic “ivory vertebra” appearance [85]. Nuclear medicine imaging with gallium demonstrates increased uptake [87]. Bone scintigraphy is nonspecific and shows avid uptake on all three phases [88]. These nuclear medicine scans can assist in metastatic workup. Ultimately, patient age, lesion location, and characteristic imaging features should raise suspicion for this diagnosis.

### 2.11. Histiocytic

#### Langerhans Cell Histiocytosis

Spinal Langerhans cell histiocytosis (LCH) represents a clonal proliferation of pathologic Langerhans-type dendritic cells. Patients can present with localized back pain, and neurological deficits may occur with vertebral collapse or epidural extension [89,90]. Vertebra plana (complete collapse of the vertebral body) is a classic presentation in children and adolescents [91]. LCH shows a slight male predominance and, although possible at any age, is diagnosed most often in childhood [92,93].

**Figure 25:**
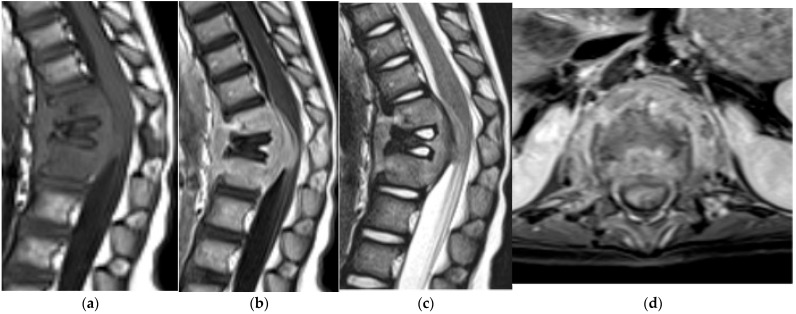
Langerhans Cell Histiocytosis. (**a**) Sagittal T1 MR, complete collapse of the L1 vertebral body (vertebra plana). Low T1 signal marrow infiltrate is present with extraosseous extension into the adjacent vertebral bodies and paraspinal soft tissues including the ventral epidural space. (**b**) Sagittal T1 post-contrast: the lesion enhances. (**c**) Sagittal T2 MR: ventral epidural extension results in severe spinal canal narrowing and compression of the conus. (**d**) Axial T1 post-contrast: extraosseous extension and enhancement are present.

Radiographs and CT typically reveal a destructive, lytic lesion involving the vertebral body, sometimes extending to posterior elements with paraspinal soft tissue extension (Figure 25) [94]. The soft tissue component may be visualized on radiographs; however, CT and MRI are the preferred modalities, not only to determine the extraosseous extent but also in evaluating any compressive complications (Figure 25). Extensive destruction of the vertebral body ultimately results in vertebra plana (Figure 25), which is more often seen in children than adults (reported incidence ~49% in one series) [94].

On MRI, lesions are usually iso- to hypointense on T1 and hyperintense on T2, with enhancement in both adults and children (Figure 25) [94]. Given that two groups of LCH include multifocal disease, bone scintigraphy and PET/CT can be beneficial if disseminated disease is suspected.

## 3. Discussion

This narrative, pictorial review summarizes the imaging appearances of primary osseous tumors of the spine across major histologic lineages, while integrating epidemiological and clinical characteristics. Because the clinical presentation of these lesions is often nonspecific, imaging plays a central role in lesion characterization (either allowing for a definitive diagnosis or narrowed differential) and subsequent clinical decision-making. In addition, a structured assessment of lesion aggressiveness (including transition zones and cortical breach), matrix composition, and marrow and canal involvement helps determine the safest and most appropriate next steps.

### 3.1. Mechanical Considerations and Fracture Risk

Spinal bone tumors must be interpreted with attention to structural integrity. Beyond their potential to exert mass effect on the spinal cord, the spine’s load-bearing function means that many tumors, both benign and malignant, can compromise spine stability, via cortical thinning, expansile remodeling, or pathologic fracture (e.g., aneurysmal bone cysts, hemangioma, and fibrous dysplasia) [95]. Accordingly, radiologic assessment must account not only for biological behavior but also for mechanical consequences, including actual or impending instability, canal/foraminal compromise, and features that could alter the urgency of care.

### 3.2. Treatment

Management is generally individualized by tumor type, location, extent, and treatment goals (e.g., curative intent, prolongation of survival, improvement of function, pain relief, prevention of fracture progression, and alleviation of spinal cord compression). Possible options range from observation to surgery, as well as radiation, systemic therapy, and interventional techniques (e.g., vertebroplasty, embolization, or lesion-directed ablation/injection) [95,96]. Decisions regarding treatment and the aggressiveness of intervention often involve consideration of established musculoskeletal tumor staging systems such as Enneking staging, which incorporates histological grade, local tumor extent, and presence of metastasis, ultimately guiding the risk–benefit assessment of potential interventions.

Despite the described utility of diagnostic imaging in characterizing primary osseous tumors of the spine, biopsy remains a key diagnostic procedure for many lesions [97]. While some lesions, such as aneurysmal bone cysts and vertebral hemangiomas, may not require biopsy due to low diagnostic yield and procedural risk [98], biopsy is critical for most lesions as tumor type and grade can have significant implications for prognosis and the extent of intervention. Imaging plays a central role in biopsy planning by identifying the safest and most diagnostically viable trajectory while ensuring treatment goals, with biopsy commonly performed using CT or fluoroscopic technique, and ensuring that the tract lies within a favorable resection plane to minimize the risk of tumor seeding in high-grade malignancy [99]. Biopsy can be non-diagnostic, particularly in sclerotic lesions, for which targeted sampling can improve yield [100]. Given the technical nuances of biopsy and complexity of management, referral to specialized musculoskeletal oncology centers for biopsy and treatment is recommended when these tumors are suspected.

### 3.3. Advanced and Adjunct Imaging

Beyond conventional CT and MRI, emerging tools can refine diagnosis in selected scenarios. Spectral/dual-energy CT can help distinguish benign from malignant lesions. Luo et al. demonstrated that quantitative spectral parameters, such as iodine density and spectral curve profiles, differentiate bone enostoses from osteoblastic metastases with high accuracy (AUC up to 0.93), outperforming conventional CT [10]. The American College of Radiology acknowledges the potential of spectral CT while noting the need for further validation [19].

Dynamic MRI, including dynamic contrast-enhanced MRI (DCE-MRI), adds physiologic and hemodynamic detail that improves tumor characterization, surgical planning, and treatment monitoring. For example, dynamic gadolinium-enhanced MRI enhances lesion conspicuity in osteoid osteoma [101]. Multiparametric approaches, such as combining intravoxel incoherent motion with DCE-MRI, have successfully differentiated atypical cartilaginous tumors from high-grade chondrosarcomas in long bones, with perfusion metrics correlating with histologic markers of angiogenesis and tumor aggressiveness [45]. These methods may have future relevance in spinal tumors.

Single-photon emission computed tomography (SPECT) is not routinely used for all primary osseous spinal tumors, but may have utility in select contexts, such as osteoid osteoma, where ^99m^Tc-MDP SPECT/CT may improve diagnostic accuracy [102].

Overall, MRI, with and without contrast, remains an appropriate first-line modality for suspected primary osseous tumors, with CT another viable option [19]. Other modalities, including dual-energy CT and SPECT, may serve non-routine, complementary roles. Continued refinement of MRI and advanced functional imaging holds the potential to further enhance diagnostic clarity and guide individualized management.

## 4. Conclusions

Primary osseous tumors of the spine are uncommon but encompass a wide spectrum of lesions with diverse biological behavior. Alongside biopsy, imaging often plays a central role in the diagnostic workup. Many tumors demonstrate characteristic CT and MRI patterns that narrow the differential diagnosis and, in some cases, allow for confident identification. Importantly, benign and malignant lesions can cause spinal instability or neural compression, making it essential that radiologic assessment evaluate mechanical integrity in addition to tumor biology. Timely, accurate imaging interpretation guides safe biopsy, informs treatment selection, and helps protect spinal and neurologic function.

## Data Availability

The original contributions presented in this study are included in the article. Further inquiries can be directed to the corresponding author.

## Abbreviations

The following abbreviations are used in this manuscript:

ABC	Aneurysmal bone cyst
BNCT	Benign notochordal cell tumor
CT	Computed tomography
DCE-MRI	Dynamic contrast-enhanced MRI
FD	Fibrous dysplasia
FDG	Fluorodeoxyglucose
LCH	Langerhans cell histiocytosis
MRI	Magnetic resonance imaging
PET	Positron emission tomography
SPECT	Single-photon emission computed tomography
VH	Vertebral hemangioma
